# Hereditary breast cancer next‐generation sequencing panel evaluation in the south region of Brazil: A novel *BRCA2* candidate pathogenic variant is reported

**DOI:** 10.1002/mgg3.2504

**Published:** 2024-08-10

**Authors:** Cesar Augusto B. Duarte, Carlos Alberto dos Santos, Cristine Domingues D. de Oliveira, Cleverton César Spautz, Laura Masami Sumita, Sueli Massumi Nakatani

**Affiliations:** ^1^ Research and Development Division Genoprimer Diagnóstico Molecular Curitiba Paraná Brazil; ^2^ Molecular Biology Clinical Laboratory Clinimol Diagnóstico Molecular São Paulo São Paulo Brazil; ^3^ Department of Surgery Hospital Nossa Senhora das Graças Curitiba Paraná Brazil

**Keywords:** BRCA2 mutation, hereditary breast cancer, next‐generation sequencing, variant interpretation

## Abstract

**Background:**

In this article, we delineate a loosely selected cohort comprising patients with a history of early‐onset breast cancer and/or a familial occurrence of cancer. The aim of this study was to gain insights into the presence of breast cancer‐related gene variants in a population from a micro‐region in southern Brazil, specifically the Metropolitan Region of Curitiba. This area exhibits a highly genetically mixed population, mirroring the general characteristics of the Brazilian people.

**Methods:**

Comprehensive next‐generation sequencing (NGS) multigene panel testing was conducted on 12 patients from the region, utilizing three different library preparation methods.

**Results:**

Two pathogenic variants and one candidate pathogenic variant were identified: *BRCA2* c.8878C>T, p.Gln2960Ter; *CHEK2* c.1100del, p.Thr367Metfs15, and *BRCA2* c.3482dup, p.Asp1161Glufs3.

**Conclusion:**

*BRCA2* c.3482dup, a novel candidate pathogenic variant, previously unpublished, is reported. The prevalence of pathogenic variants in this small cohort is similar to that described in the literature. All different library preparation methods were equally proficient in enabling the detection of these variants.

## 1 INTRODUCTION

In Brazil, breast cancer ranks as the most prevalent cancer in women across all regions, second only to non‐melanoma skin cancer. The estimated number of new cases in 2024 is approximately 70,000 (Primo, 2023). While hereditary factors contribute to less than 10% of breast cancer cases, identifying carriers of pathogenic variants associated with increased cancer risk remains a potentially cost‐effective healthcare strategy, given the high annual case volume and improved prognosis with early detection (Yoshida, 2021).

The past decade has witnessed widespread adoption of next‐generation sequencing (NGS) platforms and advanced bioinformatic tools, enabling the identification of numerous pathogenic variants in individuals with clinical presentations of hereditary breast and ovarian cancer (HBOC). The interpretation of sequence variants, guided by the American College of Medical Genetics and Genomics (ACMG) and the Association for Molecular Pathology (AMP) guidelines, relies on comprehensive databases and published literature (Richards et al., 2015).

This study emphasizes the significance of reporting pathogenic/likely pathogenic variants, including conflicting interpretations, to stimulate further discussions. Acknowledging the bias in genetic association studies toward European populations (Sirugo et al., 2019), our modest contribution aims to broaden the community's knowledge. Despite the limited sample size impacting the feasibility of future screening strategies, the study seeks to identify SNPs/Indels variants associated with breast cancer, offering potential insights for future research.

While efforts have been made to advance the diagnosis and management of HBOC in Brazil (Achatz et al., 2020), it is noteworthy that a recent comprehensive review of Brazilian germline mutations in *BRCA1* (OMIM: 113705) and *BRCA2* (OMIM: 600185) did not include patients from the specific region addressed in our study (Palmero et al., 2018). Consequently, our findings aim to fill this gap, providing accurate and actionable information to contribute meaningfully to the field.

## 2 RESULTS

The standards and guidelines for interpreting sequence variants, as outlined by the ACMG/AMP, categorize variants into pathogenic, likely pathogenic, uncertain significance, likely benign, or benign. In essence, this guideline employs a set of criteria assigned to each evaluated variant with the intention of classifying it as pathogenic or benign. The summation of these criteria values leads to the classification of a variant into one of the five tiers mentioned above. Twelve variants were identified and classified as either pathogenic (P)/likely pathogenic (LP) or variants of uncertain significance (VUS), and these are reported here.

Among the 12 patients, three P/LP variants were identified, constituting 25% of the cases. Most of the listed variants were detected in all three raw data sets generated from the library preparation methods used in this evaluation (see Table S1).

The three variants classified as either P/LP shared the commonality of a null variant effect in a gene where the loss of function is a known mechanism of disease (*BRCA2* c.3482dup, *CHEK2* c.1100del and *BRCA2* c.8878C>T). All of them met the very rare frequency criterion in population databases; variant c.3482dup was not found in any database, and to the best of our knowledge, it has never been described before.

Variant *PMS2* c.2186_2187delTGA>T was the only variant with a null variant effect in a gene where the loss of function is a known mechanism of disease that was not classified as pathogenic/likely pathogenic due to the absence of additional criteria for such. Pertinent considerations about this variant will be addressed later in the discussion section.

Other variants described here were also classified as variants of unknown significance. These variants shared a moderate level of evidence of pathogenicity, based on their absence or extremely low frequency in all databases. Additionally, they exhibited other attributes such as in silico predictions, effects on protein, and functional data that were insufficient for classification as either pathogenic or benign.

The identified variants and the respective adopted criteria are summarized in Tables 1 and 2, with references to ClinVar (National Center for Biotechnology Information, 2010) and Single Nucleotide Polymorphism database (dbSNP) (Sherry et al., 1999).

**TABLE 1:** Pathogenic/likely pathogenic variants.

Variant/NM ID	Sample ID	dbSNP	ClinVar (interpretation)	ACMG criteria	ACMG (classification)
*BRCA2* c.3482dup (NM_000059.4)	GCS003	NA	NA	Effect on Protein: PVS1 Population Data: PM2	Likely Pathogenic
*CHEK2* c.1100del (NM_007194.4)	GCS012	rs555607708	Conflicting interpretations of pathogenicity Pathogenic (48); Uncertain significance (1)	Effect on Protein: PVS1 Population Data: PM2 Reputable Source Data: PP5	Pathogenic
*BRCA2* c.8878C>T (NM_000059.4)	GCS015	rs80359140	Pathogenic	Effect on Protein: PVS1 Population Data: PM2 Reputable Source	Pathogenic
				Data: PP5	

**TABLE 2:** Variants of uncertain significance.

Variant/NM ID	Sample ID	dbSNP	Clinvar (interpretation)	ACMG criteria	ACMG (classification)
*PMS2* c.2186_2187del (NM_000535.7)	GCS007	rs587779335	Uncertain significance	Effect on Protein: PVS1 Reputable Source Data: PP5	VUS
*TOX3* c.385C>T (NM_001080430.4)	GCS003	rs201752610	NA	Population Data: PM2 In‐silico Predictions: BP4	VUS
*TSHR* c.1060G>A (NM_000369.5)	GCS003	rs200523471	NA	Population Data: PM2 In‐silico Predictions: BP4	VUS
*RAD51D* c.56T>C (NM_002878.4)	GCS005	rs1044486334	Uncertain significance	Population Data: PM2	VUS
*RAD50* c.130A>T (NM_005732.4)	GCS008	rs377388354	Uncertain significance	Population Data: PM2	VUS
*APC* c.5272_5274del (NM_000038.6)	GCS010	rs780061589	Conflicting Interpretations Of pathogenicity Uncertain Significance (3); Likely benign (1)	Population Data: PM2 Effect on Protein: PM4	VUS
*NF1* c.5825A>G (NM_001042492.3)	GCS012	rs2069601534	Uncertain significance	Population Data: PM2 Functional Data: PM1 and PP2	VUS
*SMARCA4* c.797C>T (NM_003072.5)	GCS013	rs371045201	Uncertain significance	Population Data: PM2 Functional Data: PP2	VUS
*BRCA1* c.3266T>C (NM_007294.4)	GCS014	NA	NA	Population Data: PM2	VUS

*Note*: PVS1: very strong evidence of pathogenicity, PM1, PM2, and PM4: moderate evidence of pathogenicity, PP2, and PP5: supporting evidence of pathogenicity, and BP4: supporting evidence of benign impact.

Abbreviation: NA, not applicable.

## 3 DISCUSSION

Although our NGS panel was comprehensive, it is crucial to note that the risk posed by pathogenic variants found in these genes is not evenly distributed. Gene risk stratification for HBOC has been proposed elsewhere (Petrucelli et al., 1998). For example, *BRCA1* and *BRCA2* gene mutations are responsible for the majority of HBOC cases. However, the role of *MSH6* (OMIM: 600678) and *PMS2* (OMIM: 600259) is less clear and has been a matter of dispute (Sheehan et al., 2020; Stoll et al., 2020). Ordering physicians and patients alike must be aware of the implications associated with the selected gene panel scope, including risks and actionability. With the increase in the volume of generated sequencing data over the last decades, the challenge of accurately verifying the consequences of a diverse array of variants has grown. Even with a limited gene panel sequencing, uncertainties may arise. For the two variants classified as pathogenic by ACMG criteria, the ClinVar database indicates that *BRCA2* c.8878C>T has been reviewed by an expert panel and classified as pathogenic. *CHEK2* c.1100del (*CHEK2* gene, OMIM: 604373) is listed under “conflicting interpretations of pathogenicity” in that database, although there is overwhelming reporting of pathogenicity with 48 instances, and uncertain significance reported in one instance. We understand that both variants should be reported as pathogenic. *BRCA2* c.3482dup, classified as likely pathogenic (ACMG), has not been reported in any database to the best of our knowledge, including BRCA Exchange (Cline et al., 2018), Genome Aggregation Database (gnomAD, 2023), and ClinVar. Incidentally, this variant is located in the *BRCA2* ovarian cancer cluster regions (OCCRs), where a small but statistically significant difference in the mean age at breast cancer diagnosis was found (Rebbeck et al., 2015). The mean age was greater for mutations in OCCR compared to mutations not in OCCR. This information may be relevant to the carrier of this variant, who, in our study, is described as having relatives diagnosed with breast cancer but no personal history of cancer in her 50s.

The frameshift‐caused truncated protein is rather similar to two other known pathogenic variants listed in gnomAD: variants *BRCA2* c.3481_3482dup chr13‐32337832 C>CAG p.Asp1161Glufs8 and *BRCA2* c.3487del chr13‐32337841 TG>T p.Asp1163Ilefs5, which would cause truncated proteins slightly larger than *BRCA2* c.3482dup. Consequently, we conclude that this variant should be classified as pathogenic instead of likely pathogenic. Regarding the remaining reported variants classified as VUS, we were unable to gather evidence to reclassify them as either pathogenic or benign. However, *PMS2* c.2186_2187del, a null variant in a gene where the loss of function is a known mechanism of disease, exhibits a few peculiarities worth describing. Variant *PMS2* c.2186_2187del, predicted to cause a frameshift that alters the protein's amino acid sequence beginning at codon 729 and leads to a premature stop codon 6 codons downstream, is primarily classified as VUS in ClinVar. It was found in homozygosity in this particular sample (parents' genotyping was not available). The InSiGHT (International Society for Gastrointestinal Hereditary Tumours, 2023) database also classifies this variant as uncertain, noting that the variant is likely to originate from a pseudogene. However, this does not seem to be the case for our patient since the variant and TTT, rather than TTC, at codon 751 are contained in the same aligned reads, confirming that these reads come from the *PMS2* gene, not from *PMS2CL* (pseudogene). Additional considerations were taken into account in an attempt to ascertain the classification of this variant as pathogenic or benign. On the pathogenicity front, it is noteworthy that a slightly shorter truncated protein caused by *PMS2* c.2192T>G, p.Leu731Ter, is classified as pathogenic at ClinVar by multiple submitters with no conflicts, and it aligns with ACMG guidelines. The resulting truncated protein would be very similar to that produced by c.2186_2187del, with both cases predicted to undergo nonsense‐mediated decay (NMD). A relevant publication also supports this direction; considering that variant c.2186_2187del occurs in exon 13, within a repeated dinucleotide (CTCT), it has been described that variant c.2184_2185del was detected in Turcot syndrome‐affected siblings as compound heterozygotes (R134X/2184delTC) (De Vos et al., 2004). This allelic data might suggest a variant detected in trans with a pathogenic variant for a recessive disorder (PM3). In this report, both siblings were compound heterozygotes (R134X/2184delTC), and 2184delTC was confirmed to be of maternal origin through the analysis of both parental DNAs. Considering the above, this variant could be classified as pathogenic per ACMG guidelines (PVS1, PM3, and PP5). However, conflicting evidence may challenge this conclusion.

First, *PMS2* c.2186_2187del would retain the DQHA(X)2E(X)4E motif found at the C‐terminus of the protein encoded by this gene, forming part of the active site of the nuclease. Additionally, even though the premature stop codon location is predicted to induce the mechanism of nonsense‐mediated decay (NMD), the transcripts could potentially be resistant to degradation (Maquat, 2004). In this case, the protein function could potentially remain unaffected. Second, its frequency is 2.81% in gnomAD (African), where the number of homozygotes is 14, and 1.33% in ABraOM (Naslavsky et al., 2022) (based on whole‐genome sequencing of 1171 Brazilians in a census‐based cohort); the recommended frequency threshold for *PMS2* is 0.05%. While these frequencies do not, in themselves, serve as stand‐alone evidence for benignity, they warrant further scrutiny, particularly considering the absence of information on phenotype correlation from these databases.

Further evaluation regarding the consequence of *PMS2* c.2186_2187del seems necessary, given the gene's relevance to HBOC, hereditary nonpolyposis colon cancer, and constitutional mismatch repair deficiency syndrome. Functional studies might provide additional insights into this matter. In this small sample study, the prevalence of pathogenic mutations was somewhat higher than expected, at 25%. In a much larger evaluation with comparable patient inclusion criteria, albeit involving a different ethnic population and a considerably narrower gene panel, Shao et al. (2020) found deleterious mutations at a rate of 19.50%.

As previously acknowledged, the current cohort's limited size constrains our ability to establish definitive correlations. However, the authors foresee following up on the present cohort over time, which will include longitudinal monitoring and genetic testing of family members. We are also considering expanding our cohort by enrolling more patients and their relatives. This expansion would enable us to establish a statistical correlation between the identified genetic variants and the risk of HBOC onset.

Considering all factors, the importance of identifying gene variants in breast cancer should not be underestimated. The implications of detecting a causative variant in a patient are substantial, ranging from providing an explanation for the disease to offering a proactive opportunity for family members who may be at a higher risk for the same condition.

Two variants identified in the present study in *BRCA2* (*BRCA2* c.3482dup and *BRCA2* c.8878C>T) have clear diagnostic and clinical relevance, as pathogenic variants in this gene have been proven to increase the risk of breast cancer. Additionally, these variants are associated with an increased risk of ovarian, prostate, pancreatic cancer, and melanoma. The identification of these variants would allow not only the implementation of a more intensive and early surveillance strategy, but it might also guide treatment plans. For instance, the use of poly ADP ribose polymerase (PARP) inhibitors has been shown to benefit a subgroup of patients. Additionally, oncologists may even consider bilateral mastectomy as a primary surgical treatment for breast cancer due to the elevated rate of ipsilateral and contralateral breast cancer in patients with *BRCA2* mutations (Fong et al., 2009).

Pathogenic variants in the *CHEK2* gene increase the risk of breast cancer. In particular, the *CHEK2* c.1100del variant, identified in this cohort, is associated with an estimated two‐ to threefold increased risk of breast cancer in women and a 10‐fold increased risk in men (CHEK2 Consortium, 2004; Weischer et al., 2007). This information could potentially inform discussions regarding genetic counseling for family members who are tested and found to be carriers.

## 4 MATERIALS AND METHODS

### 4.1 Ethical compliance

This project received approval from the Genoprimer Diagnostico Molecular Research Ethics Board (approval no. 011), and all individuals provided written consent for NGS testing.

Patients were loosely selected based on a previous history of breast cancer at an early age and/or a first‐degree/second‐degree relative diagnosed with cancer (specific family relationship and cancer type information might be obtained by contacting the corresponding author), as shown in Table 3. This project received approval from the Genoprimer Diagnostico Molecular Research Ethics Board (approval no. 011), and all individuals provided written consent for NGS testing.

**TABLE 3:** Basic characteristics of the study population.

Patient ID	Age range at breast cancer diagnostic^a^	Ethnicity^d^
GCS003	NA^b^	Central and Southern European
GCS005	41–45	Iberian
GCS006	51–55	Iberian
GCS007	41–45	Indigenous
GCS008	51–55^c^	Southern European
GCS009	41–45	Southern and Eastern European
GCS010	46–50	Eastern European, Iberian
GCS011	41–45	Indigenous
GCS012	26–30^c^	Iberian
GCS013	41–45	Indigenous
GCS014	56–60	Southern European
GCS015	36–40	Central and Southern European

Abbreviation: NA, not applicable.

^a^ Invasive adenocarcinoma, except GCS008 and GCS012.

^b^ No diagnosed cancer.

^c^ Ductal carcinoma in situ.

^d^ All Brazilian‐born individuals descended from indigenous families or indicated regions.

Genomic DNA was extracted from peripheral blood samples using standard methods. Gene panel library preparation was performed using three different kits: SureSelect XT HS2 DNA Reagent Kit® (Agilent, 2023), QIAseq Targeted DNA Pro Human Hereditary Breast and Ovarian Cancer Panel – PHS‐201Z‐12, QIAGEN® (Qiagen, 2023), and Twist Target Enrichment Protocol (Twist Bioscience Corp, 2023), following the manufacturer's instructions.

Pertinent data were generated in the standard order:

### 4.2 Primary analysis

FastQ files were obtained through next‐generation sequencing performed on Illumina's MiSeq System® with the Micro Kit v2 (300 cycles) flow cell.

### 4.3 Secondary analysis

Starting with paired‐end reads, FastQ files underwent secondary analysis by aligning them to the GRCh38/UCSC hg38 genome using BWA (Li & Durbin, 2009). Duplicate readings were removed, and variants (SNPs/indels) were detected with GATK HaplotypeCaller (Van der Auwera et al., 2013), generating VCF files.

### 4.4 Tertiary analysis

Variant annotation, filtering, and prioritization were carried out using “Franklin by Genoox (2022),” enabling a straightforward analysis for SNPs/indels. Data visualization was achieved through Integrative Genomics Viewer (IGV) (Robinson et al., 2023), which was crucial for reviewing data quality and reliability.

The gene panel was designed to encompass a comprehensive set of 50 genes that have been associated with HBOC, as shown in Table 4. The listed genes had their coding exon regions sequenced, extended to 10 bases from the 3′ end and 10 bases from the 5′ end.

**TABLE 4:** Genes associated with HBOC included in the panel.

*BRCA1*	*CHEK2*	*MRE11*	*SDHB*	*RINT1*
*BRCA2*	*ERBB2*	*NBN*	*SMARCA4*	*XRCC2*
*FGFR2*	*ESR1*	*STK11*	*TSHR*	*EPCAM*
*MAP3K1*	*MLH1*	*TP53*	*ABRAXAS1*	*HMMR*
*RAD51C*	*MSH2*	*RECQL*	*BLM*	*MUTYH*
*RAD51D*	*MSH6*	*XRCC3*	*DICER1*	*NQO2*
*TOX3*	*PALB2*	*NF1*	*FANCC*	*PGR*
*ATM*	*PIK3CA*	*PTEN*	*FANCM*	*PHB*
*BARD1*	*PMS2*	*APC*	*RAD50*	*POLD1*
*BRIP1*	*CDH1*	*AXIN2*	*RAD51*	*PRKAR1A*

## AUTHOR CONTRIBUTIONS

Cesar Augusto Barros Duarte and Sueli Massumi Nakatani performed the next‐generation sequencing (NGS) and bioinformatics analysis, interpreted the data, prepared the tables, and wrote and submitted the manuscript. All other authors contributed to the investigation, provided resources, and conducted the formal analysis of each case reported in this study.

## CONFLICT OF INTEREST STATEMENT

The authors declare no conflict of interest.

5

## Supporting information


Table S1.

## Data Availability

The data that support the findings of this study are available from the corresponding author upon reasonable request.
