# Supplementary figures and images for: Hereditary breast cancer next‐generation sequencing panel evaluation in the south region of Brazil: A novel BRCA2 candidate pathogenic variant is reported
Source: Mol Genet Genomic Med. 2024 Aug 10;12(8):e2504. doi: 10.1002/mgg3.2504 (PMC11316010; doi:10.1002/mgg3.2504)

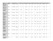

Supplement: Supplementary file 1 — Table S1. [file MGG3-12-e2504-s001.zip › preview-micro.jpg]

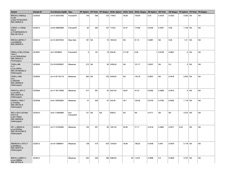

Supplement: Supplementary file 1 — Table S1. [file MGG3-12-e2504-s001.zip › preview-web.jpg]

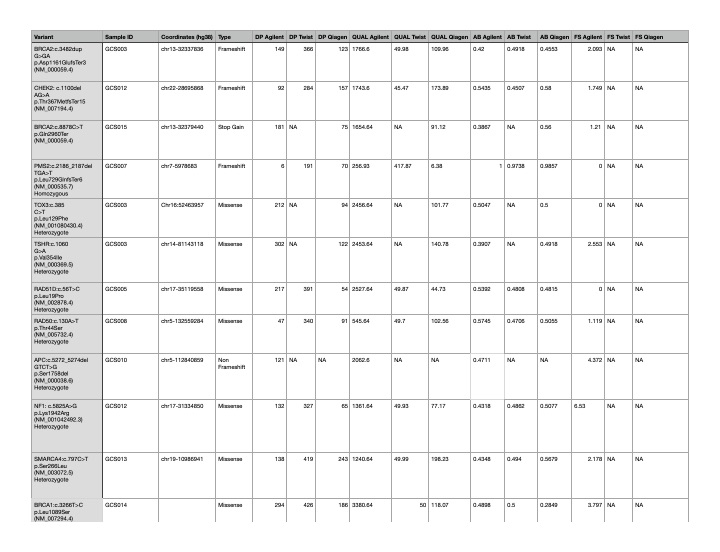

Supplement: Supplementary file 1 — Table S1. [file MGG3-12-e2504-s001.zip › preview.jpg]
